# A novel random forest approach to revealing interactions and controls on chlorophyll concentration and bacterial communities during coastal phytoplankton blooms

**DOI:** 10.1038/s41598-021-98110-9

**Published:** 2021-10-07

**Authors:** Yiwei Cheng, Ved N. Bhoot, Karl Kumbier, Marilou P. Sison-Mangus, James B. Brown, Raphael Kudela, Michelle E. Newcomer

**Affiliations:** 1 Earth and Environmental Sciences Area, Lawrence Berkeley National Laboratory, Berkeley, CA USA; 2 Statistics Department, University of California, Berkeley, CA USA; 3 Department of Ocean Sciences, University of California, Santa Cruz, CA USA; 4 Data Driven Decisions Department, Preminon LLC, Antioch, CA USA; 5 Centre for Computational Biology, School of Biosciences, University of Birmingham, Edgbaston, UK; 6 Molecular Ecosystems Biology Department, Biosciences Area, Lawrence Berkeley National Laboratory, Berkeley, CA USA; 7 Present Address: University of California, San Francisco, CA USA

**Keywords:** Ecology, Ocean sciences

## Abstract

Increasing occurrence of harmful algal blooms across the land–water interface poses significant risks to coastal ecosystem structure and human health. Defining significant drivers and their *interactive* impacts on blooms allows for more effective analysis and identification of specific conditions supporting phytoplankton growth. A novel iterative Random Forests (iRF) machine-learning model was developed and applied to two example cases along the California coast to identify key stable interactions: (1) phytoplankton abundance in response to various drivers due to coastal conditions and land-sea nutrient fluxes, (2) microbial community structure during algal blooms. In Example 1, watershed derived nutrients were identified as the least significant interacting variable associated with Monterey Bay phytoplankton abundance. In Example 2, through iRF analysis of field-based 16S OTU bacterial community and algae datasets, we independently found stable interactions of prokaryote abundance patterns associated with phytoplankton abundance that have been previously identified in laboratory-based studies. Our study represents the first iRF application to marine algal blooms that helps to identify ocean, microbial, and terrestrial conditions that are considered dominant causal factors on bloom dynamics.

## Introduction

Marine phytoplankton represent a diverse set of microorganisms that span a wide range of cell physiologies^1^, biochemical functions and ecological strategies. As key primary producers, microalgae play a crucial role in mediating the global carbon cycle and underpin food webs in oceanic and coastal environments^2,3^. Phytoplankton are responsible for ~ 50% of global primary production and net oxygen production, despite constituting less than 1% of global photosynthetic biomass^4,5^. However, when present in unusually high densities, and/or coupled with biotoxin production, harmful algal blooms (HABs) are detrimental to the environment in many ways. The frequency and magnitude of HABs have increased dramatically in the past decade and have been linked to the impacts of global climate change^6,7^. A recent study has revealed the southern California coast to be a hotspot for algal bloom formation and domoic acid (DA) production (a marine biotoxin)^8^. In 2015, record breaking concentrations of DA produced by several *Pseudo-nitzschia* species, notably the diatom, *Pseudo-nitzschia australis* bioaccumulated and poisoned coastal marine organisms, and caused the shutdown of shellfish and fish industries along the U.S. West Coast^9^. Estimated economic damages associated with blooms exceed $20 million USD per year^10^.

Current research findings point to multiple terrestrial and aquatic factors contributing to the formation of HABs in coastal environments^8,11–14^. Along the Californian coast, Ryan et al.^14^ analyzed the 2015 HAB outbreak utilizing datasets collected in the Monterey Bay region and showed that upwelling of nutrient-rich cold water, contributed to the algal blooms. However, the authors noted this condition alone was insufficient to trigger the production of DA by *Pseudo-nitzschia*. Further analysis of water chemistry revealed that low silicate to nitrate ratios reduced diatom growth, allowing DA concentration to build up within individual cells^14^. Other studies have investigated the important role of coastal watershed exports at the land–ocean interface contributing nutrients that can stimulate bloom formation^11–13^. Studies along the U.S. East Coast of Florida have pointed to elevated nitrogen and phosphorus concentrations in agricultural runoff as a major cause of these toxic algae outbreaks^15–17^, while Howard et al.^18^ reported that in southern California, wastewater effluent can provide a significant source of nitrogen to coastal waters, promoting the development of HABs.

In addition to the abiotic factors, biotic factors such as microbial community assemblage have been hypothesized to be key factors that dynamically interact with HAB. Some of these factors include the release of putative metabolites by heterotrophic bacteria that can suppress algal growth^19–21^, promotes algal growth^22^ or release remineralized nutrients from microbial degradation of algal substrates that sustains primary production^23^. Consequently, the interactions of these factors (i.e. terrestrial input, upwelling and microbial controls) are crucial for two reasons: a) they can define the overall environmental conditions that are foundational for bloom establishment, and b) co-occurring microbial assemblage may define the succession of HAB species and the fate of organic carbon transformation via remineralization. The synergistic interactions of both biotic and abiotic conditions in regulating HAB occurrences remain a key knowledge gap in phytoplankton bloom ecology.

Interactions between marine bacterial and phytoplankton communities can shape algal bloom development trajectories, impact ecosystem diversity, and modify water chemistry and have been recognized as a critical microbial loop^24^. Phytoplankton-associated bacteria break down photosynthate-released dissolved organic matter^25^ and dead algal cells^26^ and assimilate these compounds for their own growth. Bacteria, in turn, provide macro- (e.g. fixed nitrogen) and micro-nutrients (e.g. vitamin B_12_) for algal growth^27,28^. Due to such close phytoplankton-bacteria interactions, phytoplankton biomass and bacterial biomass are tightly coupled^23^. A recent study off the California Coast indicated that bacterial composition and structure are strongly influenced by phytoplankton species in blooms, and that algal biotoxin can play a role in limiting bacterial diversity^29^.

With increasing recognition of HAB problems, investments have gone into early detection and prediction of HABs through real-time monitoring of ocean, lagoon, and coastal watershed systems at regional to global scales^30^. In addition, technological advancement and proliferation of ‘big’ datasets have led to machine learning techniques as numerical tools that reveal insights into HAB dynamics and predict HAB occurrences in ways that still challenge physically-based models^31^. Recent studies explored the application of other machine-learning approaches (i.e. multiple linear regression, regression tree, support vector machine and random forest) to predict algal blooms using remote-sensing data^32,33^. Asnaghi et al.^34^ used a Quantile Random Forest to predict the concentration of the toxic benthic dinoflagellate *Ostreopsis cf. ovata* in the Ligurian Sea (North-western Mediterranean). A mathematical model predicting the occurrence of *Alexandrium minutum* in coastal waters of the NW Adriatic Sea was developed using a Random Forest (RF), which is a machine learning technique, trained with molecular data of *A. minutum* occurrence obtained by molecular PCR assay^35^. Other examples include self-organizing maps^36^ and network-based community detection approaches^37^. These RF studies identified independent controls over algal blooms and characterized their relative importance^34,35^. However, thus far, no RF analysis conducted reveals the interactive impacts of these key controls on phytoplankton bloom formation. Given that blooms follow highly non-linear pattern^38^, improvement in our understanding of relationships between bloom dynamics and interactions between key controls is warranted.

Using empirical examples, we demonstrate the utility of a novel RF algorithm, iterative random forest (iRF)^39^, in extracting stable nonlinear interactions in two algal bloom related biological scenarios in Northern California, USA. In the first example, we explore impacts of inland and marine nutrient conditions on algal abundance. In the second example, we apply iRF to a marine microbiome dataset to explore interactions between microbial community structure and phytoplankton during algal blooms. To our knowledge, this is the first application of iRF to a marine dataset that explores and identifies higher order interactions between key biological and environmental controls.

## Methods

### Iterative Random Forest

We utilized iterative Random Forest (iRF) in this study. The RF model^40^ is an ensemble-based machine learning method, where each RF includes a fixed number of Decision Trees (DT). Each model statistically learns patterns and rules using a bootstrapping technique from correlations between explanatory variables and a response variable^41^. The outputs of the trees are averaged to prevent over dependence on any single DT model and reduce the risk of over-fitting. A trained/fitted RF model is then used to predict a response variable given a set of explanatory variables. In addition, RF models also provide statistically produced measures such as permutation importance (also known as feature importance) to quantify the relative impact of the explanatory variables on the response variables. While RF is able to uncover nonlinear and linear relationships between variables, and evaluate the relative importance of the individual explanatory variables, identifying the interactions between these variables remains challenging due to the potentially intractable number of interactions^39^.

Basu et al.^39^ developed the iRF algorithm as a computationally efficient approach towards interpreting stable high order interactions between the variables in a fitted RF. Readers are referred to Basu et al.^39^ for detailed description of the iRF and applications to genomic datasets. Here we briefly describe the main iRF workflow: (1) Iteratively grow *N* number of feature re-weighted RF. The iterations are based on the Gini Importance (GI) index, which is a measure of information gain (feature importance) in each decision pathway. (2) Extract decision rules from ensemble RF outputs. Building upon the generalization of the random intersection trees algorithm (RIT), the resulting RF map from step (1) allows users to identify prevalent interactions^41^. (3) Perform an additional layer of bootstrapping to assess the stability of the recovered interactions.

### Dataset, response and explanatory variables

We provide two examples of iRF in this study. Methods and datasets used for each example are provided below.

#### *Example 1*

We explore the role of inland (terrestrial) and oceanic abiotic controls and interactions during HABs. Marine data were collected at the Santa Cruz Wharf on a weekly basis by the Central and Northern California Ocean Observing System (CeNCOOS). The dataset ranged from October 19, 2011 to December 19, 2018 (Fig. S1). Due to significant sections of missing values, observations from January 17, 2018 to December 19, 2018 were not considered. Ocean abiotic factors collected by CeNCOOS at the SCW consisted of nitrate (µM), phosphate (µM), silicic acid (Si, µM), and domoic acid (mg/L). Detailed descriptions of sampling procedures and post processing can be found in Lee and Sison-Mangus^42^ and Sison-Mangus et al.^29^. iRF was first applied to this CeNCOOS -SCW dataset using *in-situ* measurements of *chlorophyll-a* as the response variable assuming *chlorophyll-a* is a reasonable proxy for algal biomass.

Inland data were obtained from the Coastal Santa Cruz watershed (HUC8 – 180600001 – San Lorenzo-Soquel), which contain a number of rivers and creeks draining into the Pacific Ocean (Fig. S2). Water quality data were obtained from the California Environmental Data Exchange Network (CEDEN). CEDEN data were selected between January 1, 2000 to December 31, 2018, that included total ammonium, dissolved nitrate, total Kjeldahl nitrogen, total nitrogen, dissolved orthophosphate and total phosphorus, and cover the same time period of analysis as the CeNCOOS-SCW dataset mentioned above. Discharge data were obtained from the USGS National Water Information System (NWIS).

We used water quality data and discharge data from the Santa Cruz watershed to calculate flow normalized inland nutrient fluxes (FNFs) which are the mass fluxes of nutrients that reach the ocean from the San Lorenzo River (SLR) and Soquel Creek (SCk). Flow normalization is the process using the probability density distribution of discharge values in order to remove any major yearly variations of discharge. Individual contributions of SLR and SCk were historically recreated through Weighted Regressions in Time, Discharge, and Seasons (WRTDS, see details in Supplementary Information)^43^. FNF, calculated as kg/day in WRTDS, was summed for SLR and SCk on a weekly time scale, and represents the approximate watershed contributions to coastal marine water quality. WRTDS FNF estimates, and measured concentrations are provided in Fig. S3. All WRTDS FNF data were used as explanatory variables in the iRF model.

Since the main aim of Example 1 is to identify key factors and interactions among aquatic variables (terrestrial and oceanic) governing the formation of HABs, we evaluate iRF models across the oceanic, and oceanic + inland dataset. We used iRF first on the CeNCOOS data only to evaluate just the oceanic influence (oceanic CeNCOOS based SCW only), then the iRF analysis was repeated on a combined dataset using the CeNCOOS oceanic data and inland nutrient fluxes from the WRTDS methodology to evaluate the terrestrial and oceanic combined controls (CenCOOS + WRTDS Inland, SCW + Inland).

#### *Example 2*

We used a marine microbial community dataset from the Santa Cruz Wharf (SCW) in Monterey Bay (36.958 °N, − 122.017 °W), with 55 unique sampling dates and a total of 152 samples including replicate samples from April 3, 2014, to November 11, 2015 to explore abiotic and biotic interactions between prokaryotes, phytoplankton, and environmental conditions during HABs. The SCW dataset collection, description, and analysis details can be found in Lee and Sison-Mangus^42^ and Shuler et al.^44^. We used these data to explore microbial abundance patterns driven by harmful algal bloom environmental and biological drivers. We apply iRF to this microbiome dataset to: (1) identify impacts of physical, chemical and biological drivers, and (2) elucidate interactions between these drivers, on microbial abundances.

Abiotic (environmental) variables consisted of ammonium (NH_4_, µM), silicic acid (Si, µM), nitrate (N, µM), phosphate (P, µM), temperature (WTMP, ^o^C), and Domoic Acid (DA, mg/L). Biotic variables include two phytoplankton taxa represented by the dinoflagellate group *Alexandrium spp*. (Alx. Spp. cells/L) and *Pseudo-nitzschia* in the size range of the functional group *seriata* (Ps-nt. Seri. cells/L), chlorophyll-*a* (Chl-a. mg/m^3^) as a proxy for biomass, and the eleven most abundant operational taxonomic units (OTUs) from the sequence samples. *Alexandium spp*. is being monitored in the SCW because it (together with *Pseudo-nitzschia*) represents a key toxin producing algae. One missing value for both Ps-nt. Seri. and Alx. Spp. was filled in using the mean of the points before and after. The eleven OTUs include: *Octadecabacter_1, Octadecabacter_2, Euryarcheota Marine group II, Polaribacter, Flavobacteriaceae_1, Flavobacteriaceae_2, Loktanella, Cryomorphaceae, Candidatus Portiera, Idiomarina* and *Persicirhabdus*. Microbial sequences were derived from 3 μm membrane filters, suggesting the presence of both free-living and particulate-attaching microbial OTUs. Of these microbes, the taxa *Rhodobacteraceae* are known to be free-living^45,46^, while *Cryomorphaceae, Polaribacter* and *Flavobacteriaceae* are particle-associated^29,47,48^. These samples were processed using 16 s rRNA sequencing, further details on processing procedures can be found in Kempnich and Sison-Mangus^49^ and Lee and Sison-Mangus^42^. Extended SCW data descriptions can be found in Sison-Mangus et al.^29^ and data are shown in Fig. S4. Of the 274 OTUs present the eleven OTUs chosen were those with the highest counts. Before analysis, we used the Compositional Data Analysis framework, outlined in Quinn et al.^50^, to normalize the OTU data using code produced by Kempnich and Sison-Mangus^49^ which used the “zCompositions” and “compositions” R packages^51,52^. This process first modifies counts of zeros to small positives values while holding the ratios of non-zeros, converts to relative abundance, and then performs a centered log-ratio transformation.

### Modeling procedure

In each example, to determine the best performing iRF model, we trained 25 models (for Example 2, 25 models for each OTU) with randomly selected training and testing data. For Example 1, the response variable is chlorophyll-a. For Example 2, the response variables are the eleven most abundant microbial OTUs. For Example 2 due to the presence of replicates in the data, we randomly selected one replicate for each set of replicates, leaving 55 total data points. We used a stratified sampling technique to select the training/testing data, where 75 percent of the data was used as training and the remaining 25 percent held out for testing. The 95th percentile of *chlorophyll-a*, roughly two standard deviations from the mean, was used for Example 1 to target specifically bloom conditions. The median was used for Example 2 as this was the default setting for iRF and was left as is to avoid biasing the selection using any other specification.

Each model was tuned with 5 iterations, 20–30 bootstraps, and 500 trees; random intersection trees (RITs) were given a depth of 5 with 500 total trees, 2 children nodes for each RIT, and the median or 95th percentile response value, per the respective example, for leaf node threshold for converting to binary classes (class-0 and class-1). Performances of the models were determined using Nash–Sutcliffe Efficiency (Eq. 1).

1 $$ NSE = 1 - \frac{{\mathop \sum \nolimits_{i = 1}^{N} \left( {O_{i} - P_{i} } \right)^{2} }}{{\mathop \sum \nolimits_{i = 1}^{N} \left( {O_{i} - O_{mean} } \right)^{2} }} $$

where N = total number of observations, O_i_ = observations, O_mean_ = mean of the observations, and P_i_ = predictions from the iRF model.

In each example, the relative importance of the explanatory variables was evaluated using the GI for all 25 simulations. GI (a.k.a mean decrease in impurity, MDI) is a measure of variable importance that is calculated by summing the number of times a variable is used to split a node, normalized by the number of samples it splits. The higher the GI, the higher the variable importance.

Next, only the best performing model for each set of iRF models were considered for evaluation of interactions. Measures such as stability and precision of the interactions were evaluated. Stability is the proportion of bootstrap samples in which the interaction was recovered from the total number of bootstrap samples, indicating how recoverable an interaction is. Precision is the proportion of class-1 observations in leaf nodes containing the interaction, showing the degree of potential influence of the interaction on class-1 observations.

## Results and discussions

### Example 1: inland and marine controls over coastal phytoplankton abundance

**Figure 1:**
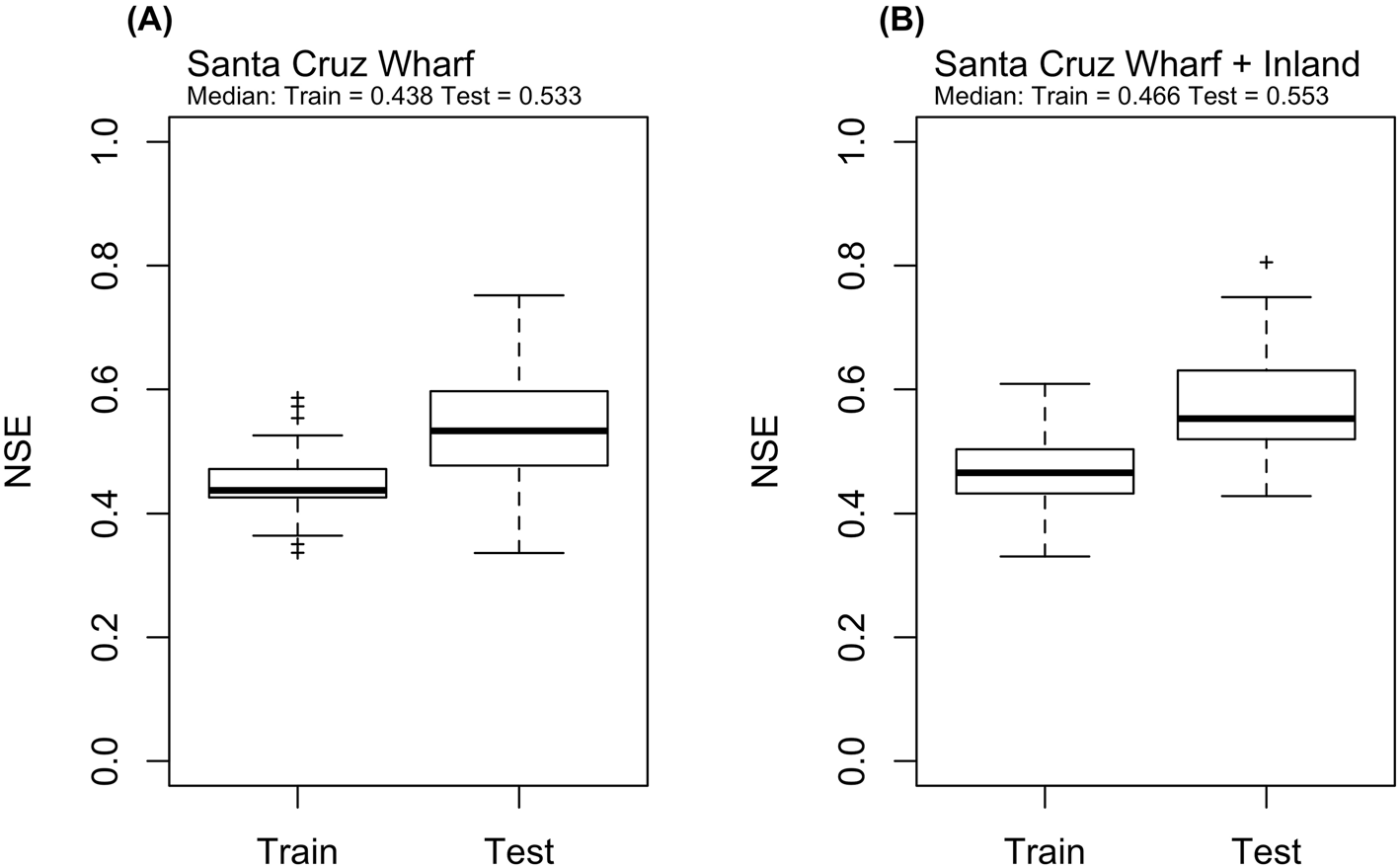
Nash–Sutcliffe Efficiencies (NSE) of the iterative random forest models when tested against training and testing data. (**A**) iRF NSE results for the Santa Cruz Wharf (SCW) only dataset, and (**B**) iRF NSE results for the SCW + inland dataset.

Observed *chlorophyll-a* showed seasonal patterns across years (2011–2018), with bloom initiation in the spring, peaking around summer and tapering off in fall (Fig. S5-Observed). Simulated (with iRF) *chlorophyll-a* captured similar seasonal trends (Fig. S5-Simulated). Utilizing only the CenCOOS-SCW dataset, the iRF models fit the training data well, with maximum and minimum NSE values at 0.59 and 0.34 respectively (Fig. 1A). When predicting the testing data, model performance is better than training, with maximum and minimum NSE values at 0.75 and 0.34 respectively. Incorporation of the inland nutrient flux (WRTDS-CEDEN) seemed to nominally improve model performance with a slight increase in median NSE (median improvement from 0.53 to 0.55 on testing data) (Fig. 1B). Maximum and minimum NSE values at training and testing (training max = 0.61, min = 0.33, testing max = 0.81 min = 0.43) are only a slight improvement respectively (Fig. 1).

**Figure 2:**
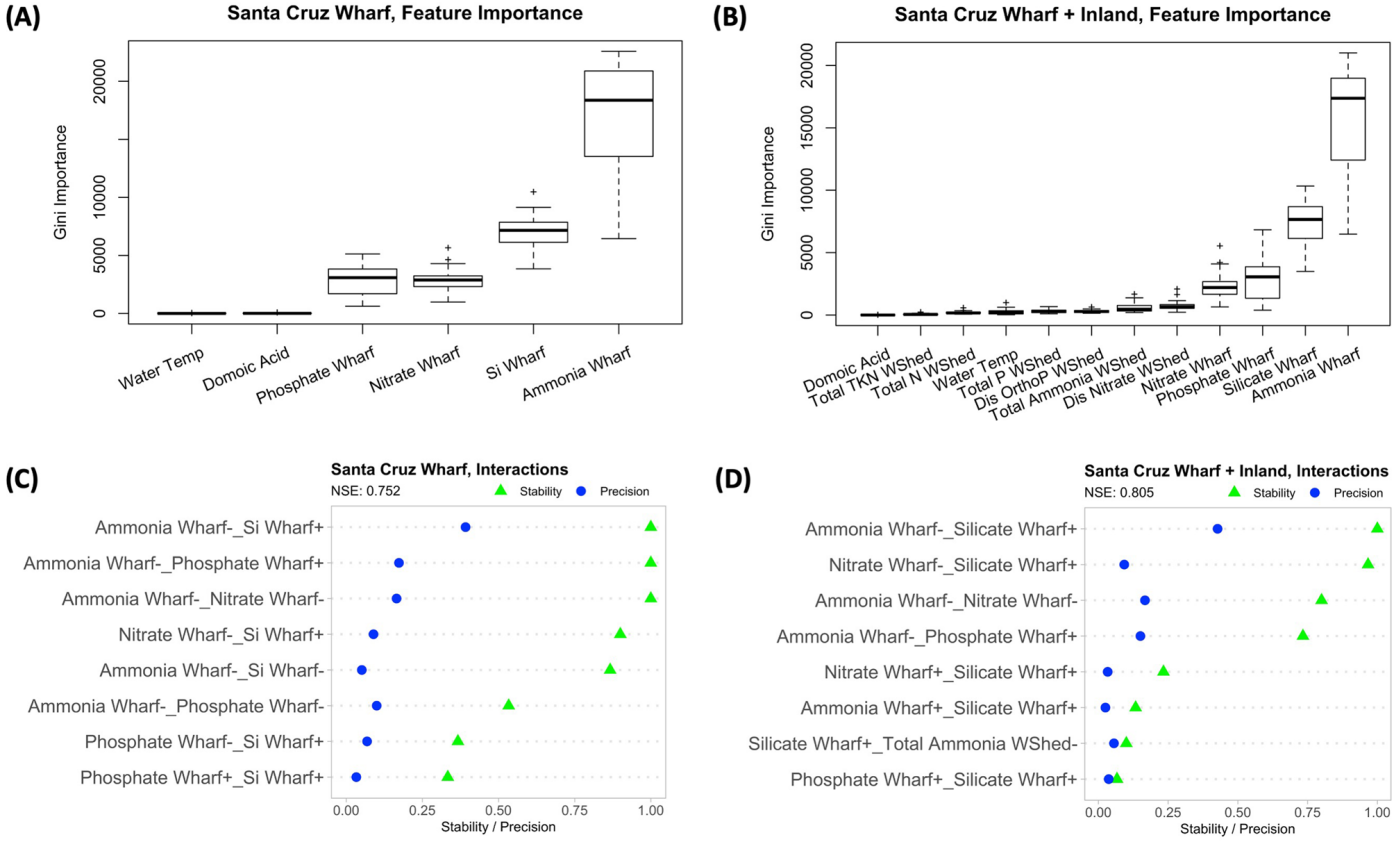
(**A** and **B**) Gini Importance of explanatory variables for iterative random forest models utilizing (left) Santa Cruz Wharf (SCW) data and (right) SCW + inland data. (**C** and **D**) The 8 most stable interactions recovered by iRF with the highest NSE utilizing (left) Santa Cruz Wharf (SCW) data and (right) SCW + inland data. Black triangles represent stability values while blue dots represent precision values.

For iRF simulations utilizing both the SCW only and SCW + inland datasets, iRF revealed the top two features to be ammonium and silicic acid concentrations at the wharf (Fig. 2a, b). For iRF analysis of SCW + inland datasets, nitrate and ammonium contributions from the watershed are less important than nutrient concentrations at the SCW (ammonium, silicic acid, phosphate and nitrate). In addition, iRF also identified stable interactions between wharf measured ammonium, silicic acid, phosphate and nitrate (Fig. 2c) despite the inclusion of inland data (Fig. 2d). In both cases, iRF consistently identified stable interactions between silicic acid and ammonium concentrations at the wharf [*sta* (ammonium(−)_silicic acid (+)) = 1.0], and silicic acid and nitrate concentrations at the wharf [*sta* (nitrate(−)_silicic acid (+)) = 0.9], suggesting the importance of the Silicate:Nitrogen ratio.

In this study, iRF highlighted the importance of nutrient control, and specifically the Silicate:Nitrogen ratio over phytoplankton biomass in Monterey Bay, similar to earlier studies that have identified coastal waters as nitrogen limited^53,54^. Springtime upwelling of nutrient rich water into the bay can jumpstart phytoplankton growth, with most of the bloom retained within the bay. Analysis of average nutrient profiles in the Monterey Bay region for April–June (1993–2016) indicate high Silicate:Nitrate ratios^14^ which can enhance productivity when concentrations are also high. However, when the Silicate:Nitrogen ratio is lowered, as in the case of spring to summer of 2015, this condition contributed to the highest recorded production of DA^14^. The form of nitrogen is also important in controlling growth and toxic production. Studies have shown that ammonium contributes to greater toxin production than nitrate in the marine dinoflagellate *Alexandrium*^55,56^, while diatom toxin production can be enhanced with addition of urea^12,57^.

Onsets of coastal HABs have been attributed to both oceanic and inland watershed factors. Along the California coast, studies suggest that seasonal upwelling of nutrient-rich cold water coupled with anthropogenic nutrient inputs (e.g., agriculture, urbanization) from watersheds have contributed to algal blooms^10–13,18^. In our study, we found the inclusion of watershed derived inland dissolved nutrient data nominally (but consistently) improved model performance when compared against simulations utilizing only the oceanic SCW data. However, we emphasize that watershed nutrient fluxes were not identified as a dominant control despite indications from prior studies that terrestrially derived nutrients should be a factor. While upwelling events mostly provide ample dissolved nutrients to fuel algal growth in Monterey Bay, watershed exports of dissolved nutrients (particularly N) can also contribute to phytoplankton growth in the bay^58,59^. Previous studies have identified the coastal Pajaro River draining the San Lorenzo-Soquel watershed as a key predictive feature on blooms during the fall and winter^58^. In this study, the San Lorenzo and Soquel rivers (known for lower nutrient loadings than the Salinas and Pajaro rivers) were chosen for their proximity to SCW. Lane et al.^58^ also identified oceanic *chlorophyll a* and silicic acid as key predictive variables on yearly time-scales, which suggests there is an important trade-off in nutrient sources to algal growth depending on the time of year.

Our results help to reveal two key pieces of information about the importance of oceanic versus terrestrially derived nutrients: (1) on the Pacific Coast, inland nutrient fluxes may be more relevant during periods when contributions from upwelling are less significant. Our work did not include oceanic upwelling variables, such as the Bakun index or the recently developed Coastal Upwelling Transport Index (CUTI) and Biologically Effective Upwelling Transport Index (BEUTI), and we suggest future studies utilize this as an indicator^60,61^. Other confounding sources of nutrients include groundwater discharge to coastal zones^59,62,63^. (2) iRF models such as ours can help reveal which oceanic and terrestrially derived variables are important contributing factors to blooms. Other watersheds contributing to coastal zones (e.g. Great Lakes, Delaware Bay, Florida) have been implicated for their outsized roles on blooms (e.g. Howard et al.^18^). In southern California, rainfall patterns have been suggested as an important factor impacting algal bloom dynamics^64^ because storm water discharge brings greater than 95% of the annual runoff from coastal watersheds into coastal ecosystems^65^. As a result, freshwater inputs can have a major impact on density stratification and nutrient levels. With nitrogen loading projected to increase through climate-induced precipitation by 19% for the major river systems in the US^17^, these coastal systems may be shifting towards more inland control with time. Whether or not watersheds play a role, the magnitude of that role, and how coastal exports will change in the future is important because HAB management strategies often point to watersheds as the origin of nutrients associated with blooms, and quantitative information will help to close this costly decision-making gap. Future work will assess a larger and more dynamic range of coastal contributions.

### Example 2: interactions between phytoplankton, microbial communities, and abiotic factors

**Figure 3:**
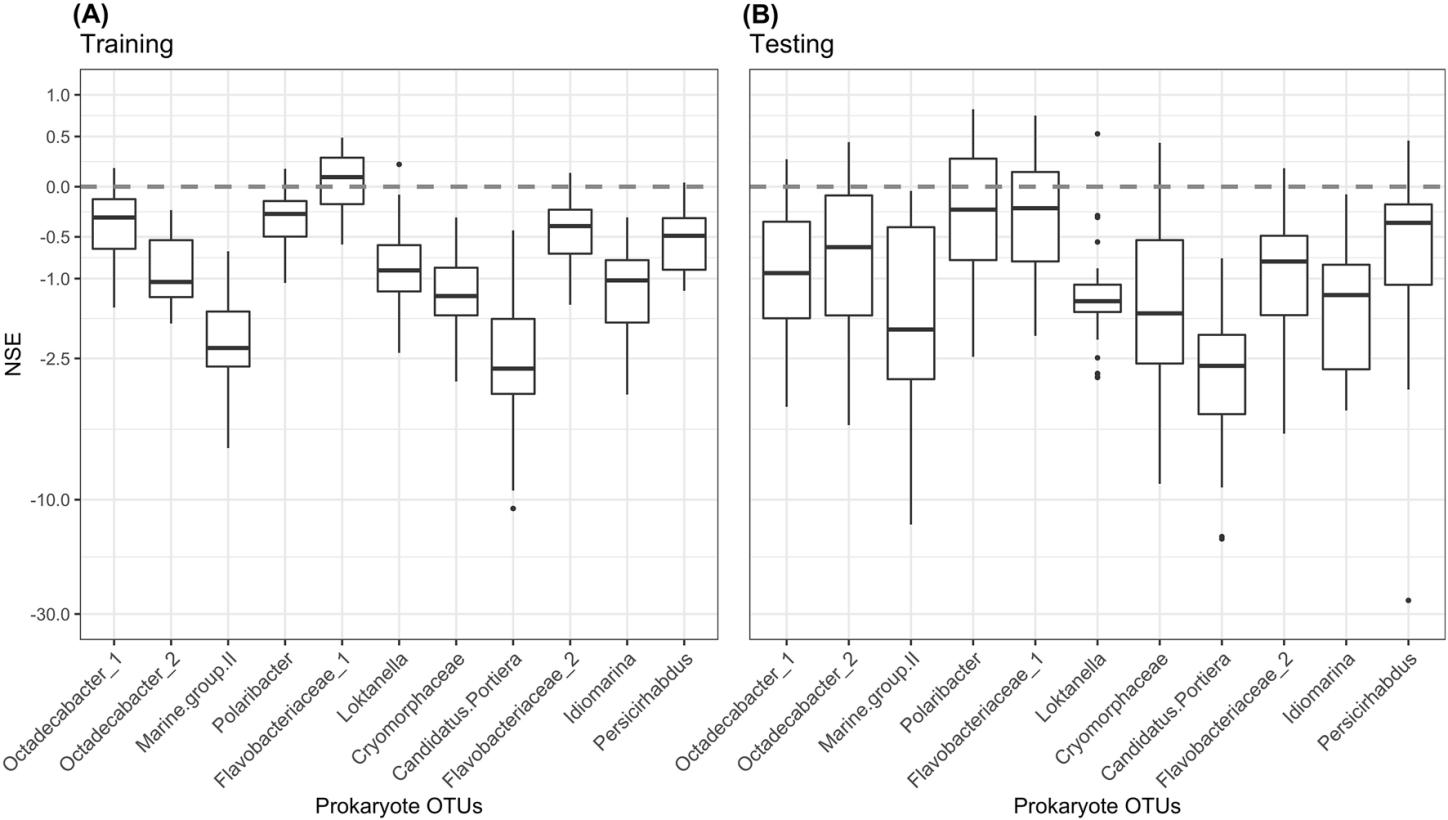
Nash–Sutcliffe Efficiencies (NSE) of the iterative random forest models when tested against (**a**) training and (**b**) testing data subsets of microbial OTUs that are part of the marine microbiome dataset collected from Santa Cruz Wharf. X-axes are the microbial strains and the y-axes are the NSE values.

In Example 2, we apply iRF to elucidate interactions between environmental and phytoplankton drivers (explanatory variables) on microbial abundances (response variables) to explore the dynamics between HABs and bacteria. We emphasize that this methodology is to identify interactions between variables (phytoplankton-bacteria interactions) and not necessarily to predict one variable or another. During both training and testing, performance of iRF models as measured by Nash–Sutcliffe Efficiencies (NSE) were found to have notable differences between microbial OTUs (Fig. 3). We evaluated the interactions in the top performing model for each OTU. Although an NSE > 0.65 is the benchmark value that earlier studies have identified as “acceptable” for model performance, during testing we found that only *Polaribacter* and *Flavobacteriaceae* produce models above this threshold^66^. Further, models do not always perform well as shown by simulations with NSE values below 0 (Fig. 3). The maximum NSE value is 1.

**Figure 4:**
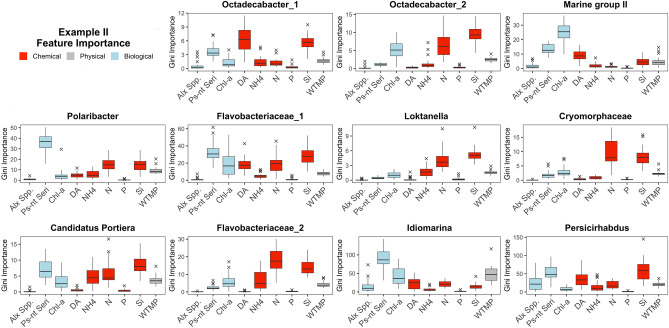
Feature importance of explanatory variables for each bacterial OTU. X-axes are the explanatory variables. The explanatory variables are categorized and color coded: red – chemical, grey – physical, blue – biological. Biotic (environmental) ocean measures consisted of ammonium (NH_4_, µM), silicic acid (Si, µM), nitrate (N, µM), phosphate (P, µM), temperature (WTMP, °C), and Domoic Acid (DA, mg/L). Biotic measures include *Alexandrium spp*. (Alx. Spp. cells/L), *Pseudo-nitzschia* in the size range of the functional group *seriata*, (Ps-nt. Seri. cells/L), and chlorophyll-*a* (Chl-a. mg/m^3^) as a proxy.

We evaluated the GI index of explanatory variables for each microbial OTU in this study (Fig. 4). iRF analysis revealed one or more of the biological variables (i.e. *Alexandrium spp*., *Pseudo-nitzschia seriata*, chlorophyll-a) to be dominant interacting drivers on the abundances of the microbial OTUs: *Octadecabacter* (1 and 2), *Flavobacteriaceae* (1),* and Marine Group II.* In particular, *Pseudo-nitzschia seriata class* consistently remained a key driver to the microbial OTUs belonging to the *Octadecabacter* genera (*Rhodobacteraceae*), an important bacterial group that participates in marine biogeochemical cycling and biofilm development^67,68^, and has been associated with blooms^69^. Similarly, the *Polaribacter* OTU, a *Bacteroidetes* genera, is highly associated with *P. seriata* and nitrogen, as similarly seen during *Pseudo-nitschia* spring blooms in San Pedro Bay, USA^70^. *Alexandrium spp* was identified as a key driver to an OTU belonging to *Flavobacteriaceae* families. It should be noted that *Alexandrium spp* is usually a minor component of the phytoplankton assemblage at SCW, and as such, this result may actually be an indicator of dinoflagellate influence. Additionally, iRF analysis revealed chemical species (i.e. silicic acid and ammonium) as key drivers for the remaining four OTUs (*Polaribacter, Candidatus Portiera, Loktanella, Flavobacteriaceae 1,* and *Persicirhabdus*). Previous analysis of this dataset showed silicic acid to be associated with abundance for some OTUs^44^.

Stable interactions identified by iRF (Table 1) point to roles of the microbial OTUs at different stages of the bloom events. For each microbial OTU, we used the iRF model with the highest NSE to further elucidate stable interactions between key explanatory variables (Table 1). Measures of stability and precision of the interactions are evaluated and compared in Table 1. In Table 1, stabilities for most interactions are 1 or near 1, thus only precision is shown in the table. Stable interactions as identified through iRF analysis of the dataset are consistent with previously observed microbial-phytoplankton-environmental interactions^20,25,26,71–73^.

**Table 1:** The 5 most stable interactions recovered by iRF with the highest NSE during prediction for each OTU.

Octadecabacter 1	NSE: 0.266	Octadecabacter 2	NSE: 0.440	Marine group II	NSE: − 0.040		
Interaction	Precision	Interaction	Precision	Interaction	Precision		
**Example II top 5 interactions**							
DA+_Ps-nt Seri−	0.787	N−_Si−	0.796	Chl-a−_Ps-nt Seri+	0.852		
DA+_Si−	0.754	Chl-a+_Si−	0.731	Chl-a−_WTMP−	0.800		
Ps-nt Seri−_Si−	0.586	Si−_WTMP+	0.727	Chl-a−_DA+	0.770		
DA−_Si−	0.523	Chl-a+_N−	0.634	Chl-a−_DA−	0.532		
DA+_Ps-nt Seri+	0.690	N-_WTMP+	0.625	Chl-a−_Ps-nt Seri−	0.531		

Polaribacter 2	NSE: 0.813	Flavobacteriaceae 1	NSE: 0.736	Loktanella	NSE: 0.529	Cryomorphaceae 2	NSE: 0.434
Interaction	Precision	Interaction	Precision	Interaction	Precision	Interaction	Precision
Ps-nt Seri+_WTMP−	0.684	Ps-nt Seri+_Si−	0.766	Si−_WTMP+	0.698	N−_Si−	0.751
N+_Ps−nt Seri+	0.666	Si−_WTMP-	0.725	N−_WTMP+	0.684	Chl-a−_Si−	0.716
Ps-nt Seri+_Si+	0.647	N−_Ps-nt Seri+	0.707	N−_Si-	0.663	Si−_WTMP−	0.702
N−_Ps−nt Seri+	0.643	N−_WTMP−	0.664	N−_NH4−	0.638	Chl-a+_Si−	0.667
DA−_Ps-nt Seri+	0.611	N−_Si−	0.571	NH4−_Si−	0.590	Ps-nt Seri+_Si−	0.846

Candidatus Portiera	NSE: − 0.742	Flavobacteriaceae 2	NSE: 0.178	Idiomarina	NSE: − 0.073	Persicirhabdus	NSE: 0.457
Interaction	Precision	Interaction	Precision	Interaction	Precision	Interaction	Precision
NH4−_Si−	0.674	N−_Si−	0.893	Chl-a−_Ps-nt Seri−	0.617	Ps-nt Seri+_WTMP+	0.875
NH4−_Ps-nt Seri−	0.646	N−_NH4−	0.849	Chl-a−_WTMP+	0.596	Ps-nt Seri+_Si−	0.861
Chl−a+_Si−	0.575	Chl−a+_N−	0.703	N+_Ps−nt Seri−	0.577	NH4−_Si−	0.815
Ps-nt Seri−_Si−	0.559	Chl-a−_N−	0.662	Alx Spp. −_Ps-nt Seri−	0.552	NH4−_Ps-nt Seri+	0.767
Chl−a+_Ps-nt Seri−	0.550	N−_Ps-nt Seri−	0.762	Ps-nt Seri−_WTMP+	0.551	Alx Spp.+_Ps-nt Seri+	0.760

The direction of change is indicate by the + or the − sign.

Specifically, we find stable interactions relating to high phytoplankton abundance (bloom increase) in the following two OTUs (with NSE greater than 0.65): *Flavobacteriaceae_1* and *Polaribacter*. *Flavobacteriaceae_1* abundances are related to the interaction of increasing *Pseudo-nitzschia seriata,* silicic acid and nitrogen *(Flavobacteriaceae_1,* first and third interactions in Table 1)*. Polaribacter* abundances are related to the interaction of increasing *Pseudo-nitzschia seriata,* silicic acid and nitrogen (*Polaribacter,* second to fourth interactions in Table 1). This assemblage of OTUs may have developed host-specific interactions with *Pseudo-nitzschia seriata* as shown in a recent study on *Pseudo-nitzschia-microbiota* association^20^. These interactions highlight the important role of microbial OTUs during bloom events*. Flavobacteriaceae* have been found to be abundant during blooms associated with diatoms or dinoflagellate^70,74–76^. The family *Flavobacteriaceae* has been recognized for their important roles in the microbial loop in coastal environments^71,72^ due to their ability to breakdown high molecular weight photosynthate-released organic compounds^26,77^ and dead algal cells^26^.

Results from iRF analysis point to several potential future studies that may better decipher the ecological functions or algal-specific associations of various bacterial groups. Controlled in vitro experiments can be conducted to elucidate specific bacteria-phytoplankton (e.g. Flavobacteraceae- phytoplankton) physical interactions, bloom formation, toxin production, and the associated consequences on nutrient (e.g. coastal carbon) cycling. Future field studies should also consider micro-nutrient measurements (e.g. iron). *Idiomarina* (one of the OTUs investigated in this study) has been characterized as a siderophore-producing bacteria that enhances microalgal growth under iron deficiency^78^. However, no iron data were available for iRF analysis in this study. Future numerical modeling studies of interacting phytoplankton-bacterial communities can also help quantify fluxes exchanged within the community and with the environment, and simulate growth. Future analysis can also focus on elucidating the important interacting and mediating effects of microbial OTUs on coastal nutrient and element dynamics and how these effects either favor or limit HABs (i.e. conditions favoring dinoflagellate/diatom HABs).

## Conclusions

In this study, the novel iterative random forest (iRF) model was applied to two algal bloom related cases along the California coast to identify key governing factors and stable interactions surrounding: (1) phytoplankton abundance in response to coastal conditions and inland nutrient fluxes, and (2) microbial abundance and harmful algal bloom environmental and biological conditions. Our study represents the first such iRF application to marine algal blooms. Further we utilized iRF to elucidate stable interactions between key drivers. In the first case, iRF helped reveal that in Monterey Bay, inland nutrient fluxes may be more relevant during periods when contributions from upwelling are less significant. Given the major inter-annual variability in upwelling and precipitation conditions along the Pacific Coast from climate oscillations (e.g. El Niño Southern Oscillation), the strong variability in watershed versus oceanic drivers is an area of future research. In the second case, iRF identified microbial abundance patterns associated with algal bloom ecology. Specifically, we found a quantifiable stable interaction related to algal blooms between *Pseudo-nitzschia* and *Polaribacter* and *Flavobacteraceae* OTUs. The dynamics between these algal-microbial interactions and the surrounding abiotic environment will require future studies to better decipher the ecological functions, abiotic interactions, and algal-specific associations of these bacterial OTUs.

## Supplementary Information

Supplementary Information.
