# Supplementary material for: A novel random forest approach to revealing interactions and controls on chlorophyll concentration and bacterial communities during coastal phytoplankton blooms
Source: Sci Rep. 2021 Oct 7;11:19944. doi: 10.1038/s41598-021-98110-9 (PMC8497483; doi:10.1038/s41598-021-98110-9)
Supplement: Supplementary file 1 — Supplementary Information. [file 41598_2021_98110_MOESM1_ESM.pdf]

A Novel Random Forest Approach to Revealing Interactions and Controls on Chlorophyll Concentration and Bacterial Communities During Coastal Phytoplankton Blooms

Yiwei Cheng,[1,*] Ved N. Bhoot,[1], Karl Kumbier,[2,^], Marilou P. Sison-Mangus[3], James B. Brown,[2,4,5,6] Raphael Kudela[3], and Michelle E. Newcomer[1]

[1]Earth and Environmental Sciences Area, Lawrence Berkeley National Laboratory, Berkeley, CA, USA

[2]Statistics Department, University of California, Berkeley, CA, USA

[3]Department of Ocean Sciences, University of California, Santa Cruz, CA, USA

[4]Data Driven Decisions Department, Preminon LLC, Antioch, CA, USA

[5]Centre for Computational Biology, School of Biosciences, University of Birmingham, Edgbaston, United Kingdom

[6]Molecular Ecosystems Biology Department, Biosciences Area, Lawrence Berkeley National Laboratory, Berkeley, CA, USA

*Correspondence: yiweicheng@gmail.com

[^]now at: University of California, San Francisco, CA, USA

Supporting Information

3 Pages

5 Figures

Additional Background

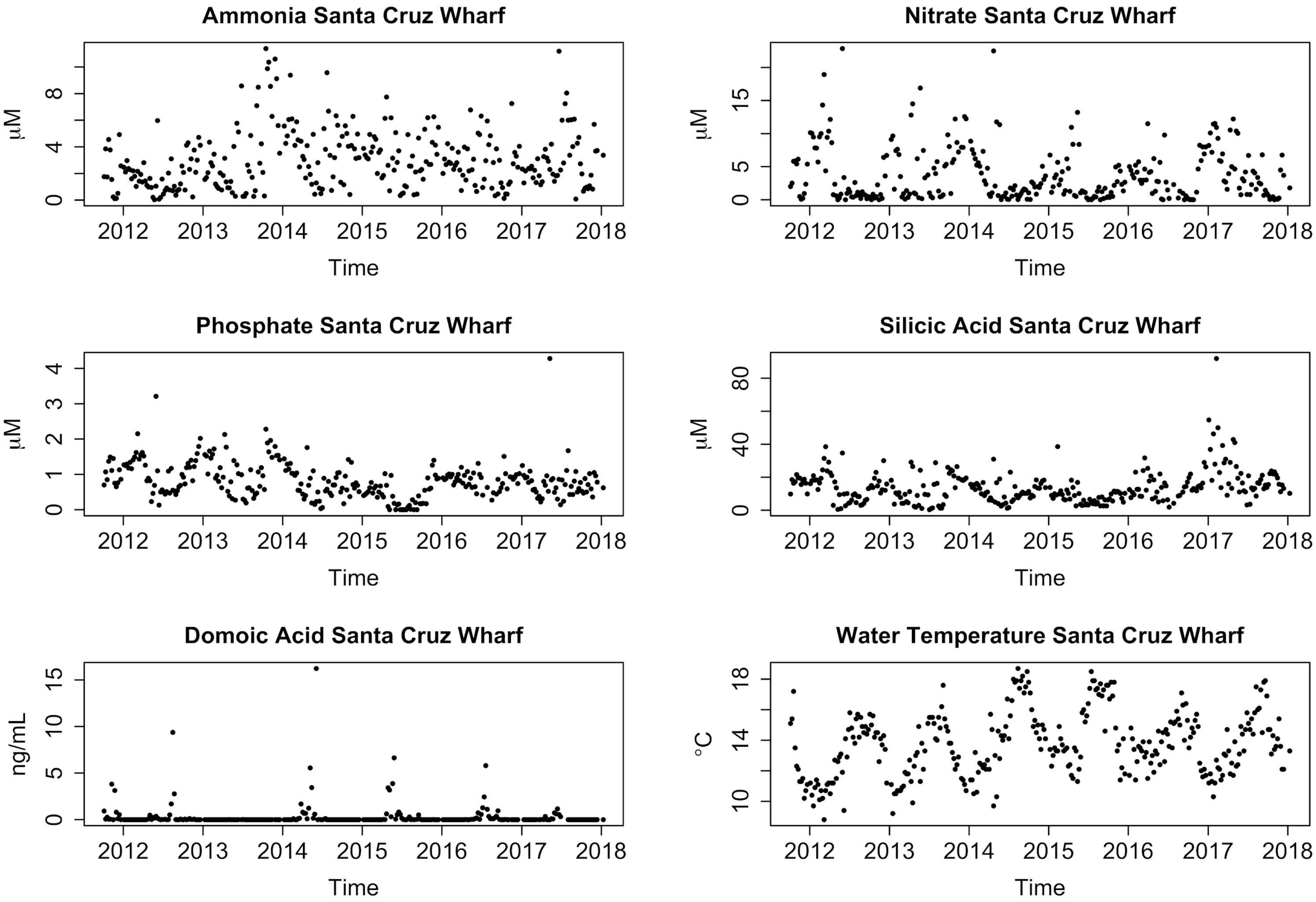

Figure S1: Chemical and physical variables recorded at Santa Cruz Wharf (CeNCOOS) which includes Ammonia, Nitrate, Phosphate, Silicic Acid, Domoic Acid, and Water Temperature.

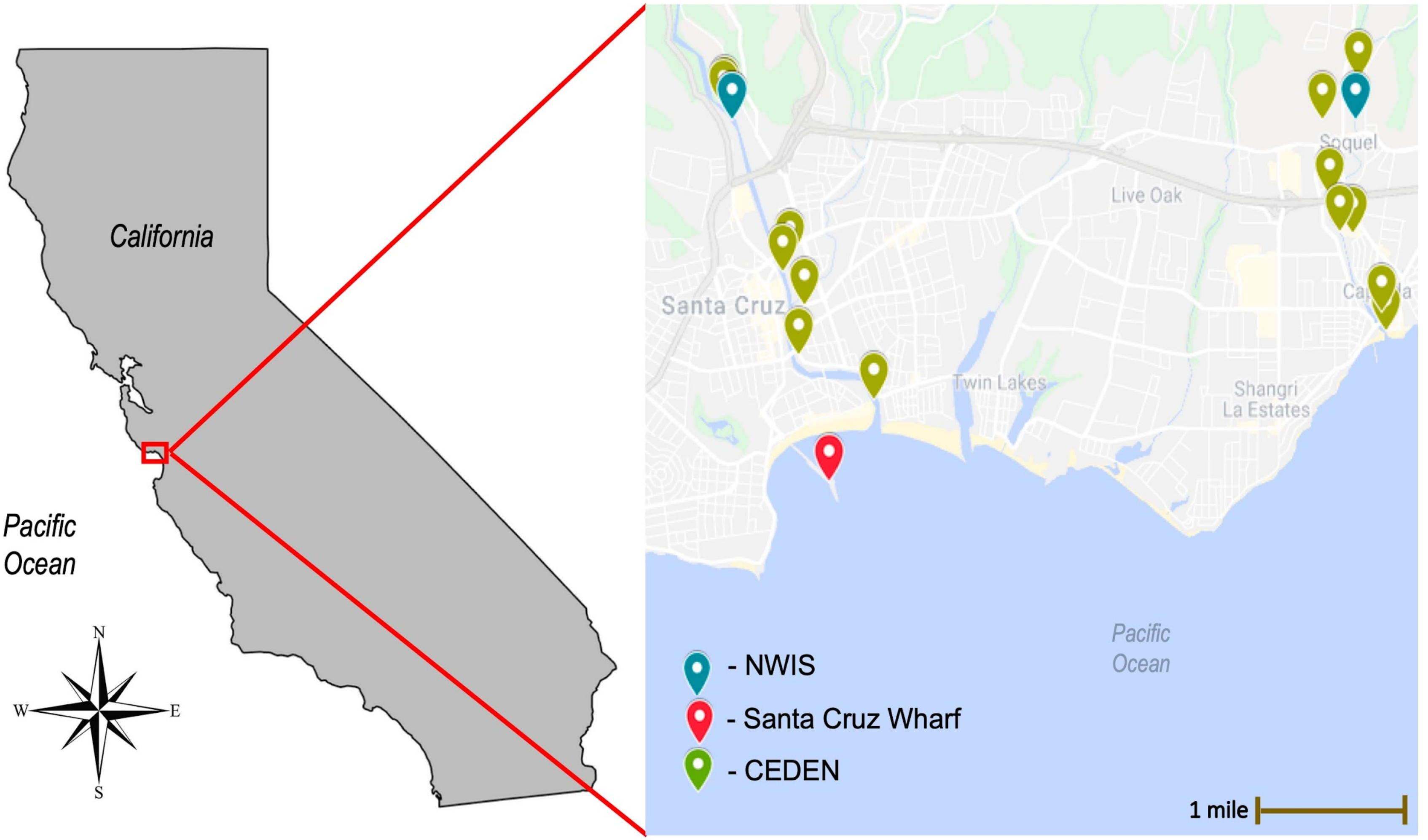

Figure S2: California Environmental Data Exchange Network stations (green markers), National Water Information System stations (blue markers), and Santa Cruz Wharf location (red marker). River directly right of SC Wharf is the San Lorenzo River and the far east water body with observation stations is Soquel Creek (Google Maps).

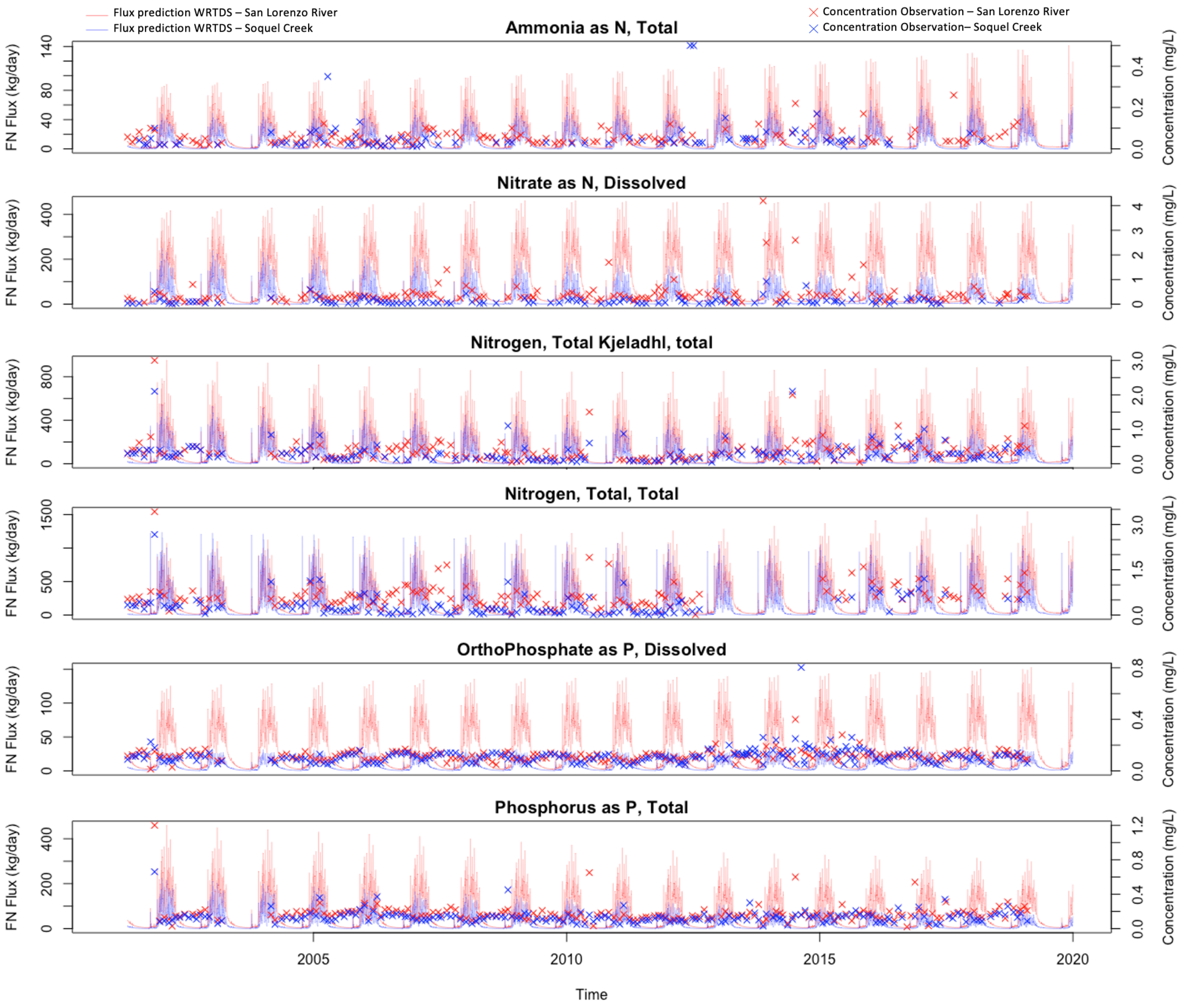

Flux prediction WRTDS – San Lorenzo River
Flux prediction WRTDS – Soquel Creek
Concentration Observation – San Lorenzo River
Concentration Observation– Soquel Creek
Ammonia as N, Total
Nitrate as N, Dissolved
Nitrogen, Total Kjeladhl, total
Nitrogen, Total, Total
OrthoPhosphate as P, Dissolved
Phosphorus as P, Total
FN Flux (kg/day)
Concentration (mg/L)
2005
2010
2015
2020
Time

Figure S3. Inland nutrients used for Example 2 (SCW + Inland) produced using data collected from CEDEN observations (mg/L, blue and red points) and the historical inland water quality recreation with WRTDS (kg/day, blue and red lines) using the EGRET package in R (Hirsch and De Cicco 2015; R Core Team 2021).

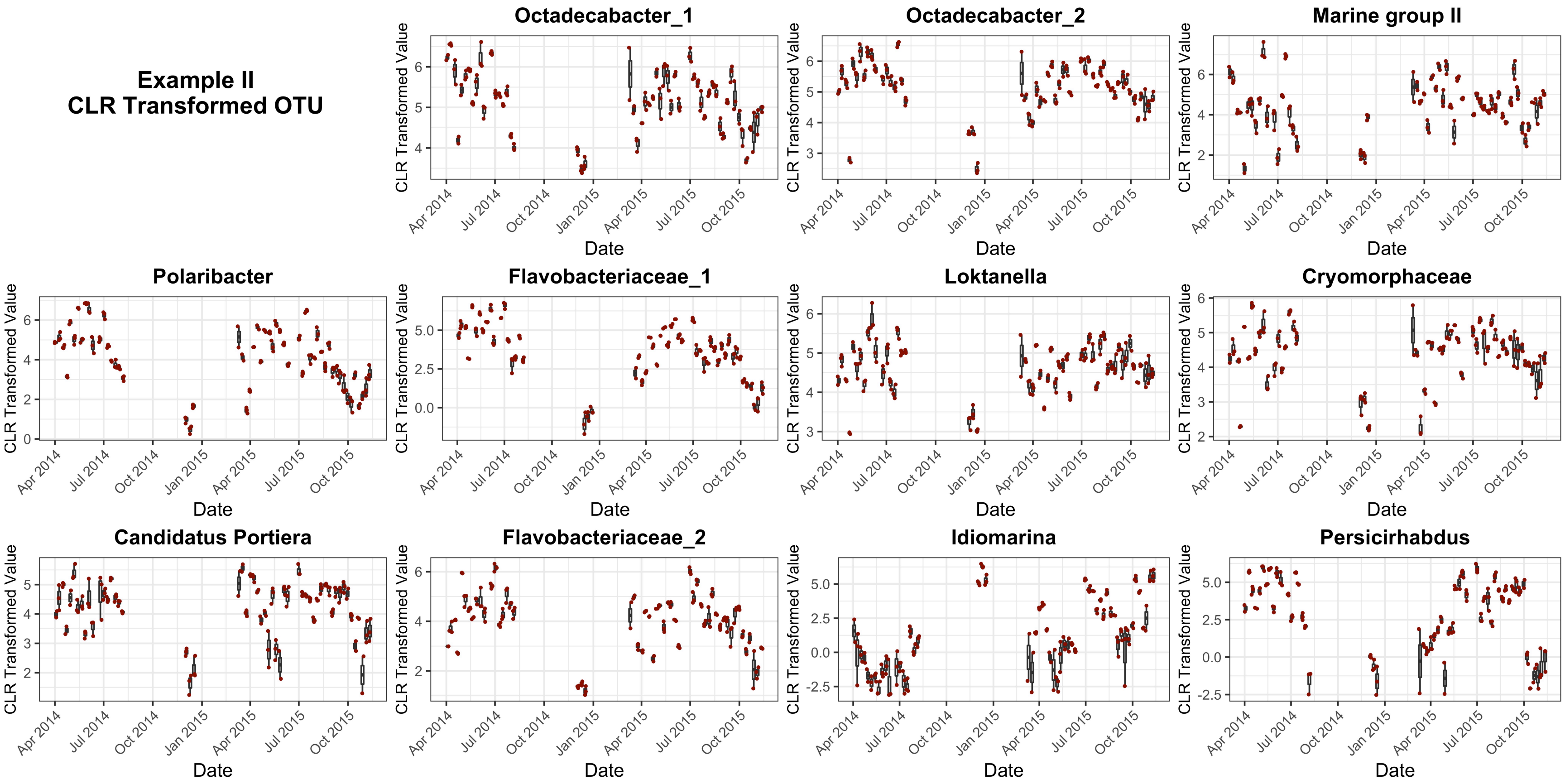

Figure S4. CLR transformed values over time for each bacterial OTU.

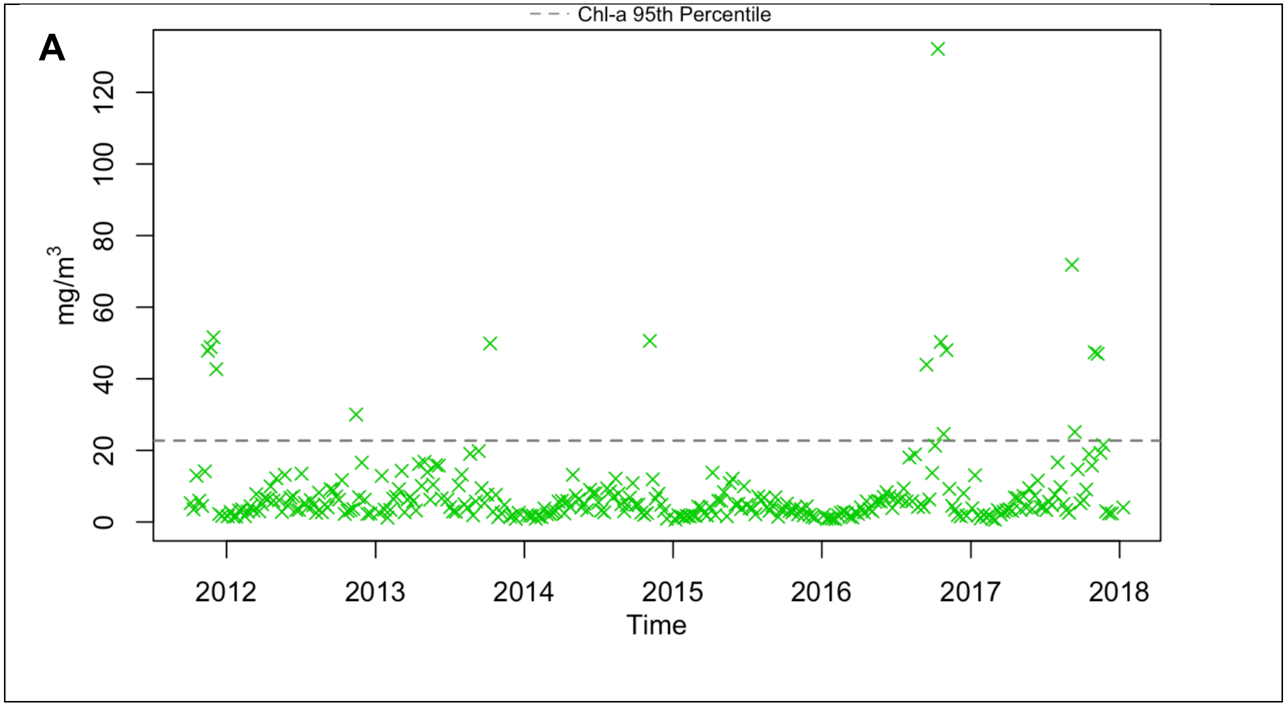

Chl-a 95th Percentile
A
mg/m³
0
20
40
60
80
100
120
2012
2013
2014
2015
2016
2017
2018
Time

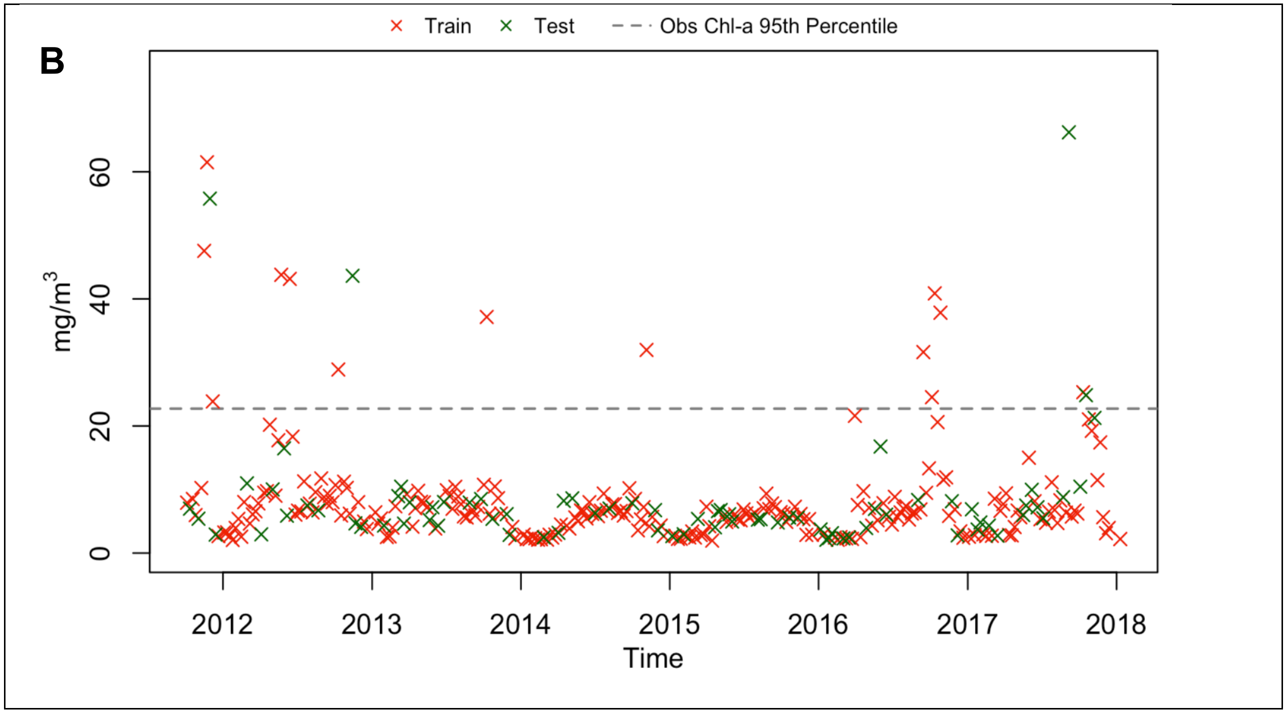

Figure S5. Time series of observed (A) and simulated (B) chlorophyll a at Santa Cruz Wharf using SCW + Inland dataset (CeNCOOS-CEDEN).

Additional Background

Weighted Regressions on Time, Discharge, and Seasons (WRTDS) Implementation on CEDEN Data. WRTDS is a statistical method to assess long timescale trends of water quality data. By breaking down water quality data trends into trend, season, response from discharge, and random variability, WRTDS estimates historical concentration of inputted water quality. WRTDS was implemented using the EGRET package in the R language.[1] Necessary data parameters for WRTDS include, daily minimum concentration, daily maximum concentration, whether a daily recording is uncensored or not, and the average concentration for the day. Due to instrument uncertainty, reporting limits are used in water quality measurements. A reporting limit is the threshold where the precision and accuracy of sample detection and measurement worsens. To simplify the process for formatting the CEDEN data to EGRET specifications, a procedure for keeping and discarding data was created to take into account cases for days with only censored measurements and those with a mix of censored and uncensored. For cases with censored only within a day, the difference between the respective reporting limits for each data point and its measurement was taken. Of the set of differences, the data point with the smallest difference was kept. The daily minimum for that day was then set as NA, the daily mean as the measured value for the kept data point, the daily maximum as the reporting limit, and “uncen” as 0. For cases with both uncensored and censored data, all censored data was discarded. Of those points kept, the daily minimum was set as the minimum measurement, the daily mean as the mean measurement, the daily maximum as the maximum, and “uncen” as 1. After formatting was

finished, complete records of daily discharge stations from the National Water Information System (NWIS) were located within Santa Cruz watershed (San Lorenzo River Station – 11161000, Soquel Creek Station – 11160000). WRTDS was then implemented and each analyte's daily history of concentrations, fluxes, and flow normalized versions of the two were produced.

Reference

1. Hirsch, R.M., and De Cicco, L.A., 2015, User guide to Exploration and Graphics for RivEr Trends (EGRET) and dataRetrieval: R packages for hydrologic data (version 2.0, February 2015): U.S. Geological Survey Techniques and Methods book 4, chap. A10, 93 p., doi:10.3133/tm4A10
2. R Core Team (2021). R: A language and environment for statistical computing. R Foundation for Statistical Computing, Vienna, Austria. https://www.R-project.org/.
